# Use of Tissue Specimens from Stereotactic Biopsies for Patient-Derived GBM Organoid-Based Drug Testing

**DOI:** 10.3390/cells14100701

**Published:** 2025-05-12

**Authors:** Amélie Wöllner, Adrian Paul, Maddalena Arquilla, Junguo Cao, Catharina Lotsch, Gerhard Jungwirth, Lena Jassowicz, Andreas von Deimling, Andreas W. Unterberg, Sandro M. Krieg, Martin Jakobs, Rolf Warta, Christel Herold-Mende

**Affiliations:** 1 Division of Experimental Neurosurgery, Department of Neurosurgery, Medical Faculty of Heidelberg, University of Heidelberg, 69120 Heidelberg, Germany; 2 Department of Neuropathology, Medical Faculty of Heidelberg, University of Heidelberg, 69120 Heidelberg, Germany; 3 Division for Stereotactic Neurosurgery, Department of Neurosurgery, Medical Faculty of Heidelberg, University of Heidelberg, 69120 Heidelberg, Germany

**Keywords:** glioblastoma, patient-derived tumor organoids, stereotactic biopsy, precision medicine

## Abstract

*IDH*-wildtype glioblastoma (GBM) represents the most common malignant form of brain tumor and is still incurable despite comprehensive therapeutic efforts. Due to tumor location and patient condition, open surgical resection of recurrent GBM is not always feasible. In these cases, frame-based stereotactic biopsies represent a less invasive technique to obtain tissue samples for diagnostics. However, whether this material would also be sufficient to prepare tumor organoids (TOs) and perform drug screenings has not been addressed so far. In this study, we present our highly optimized workflow for generating standardized patient-derived GBM TOs from single-cell suspensions using limited biopsy-derived material. We highlight crucial steps within the procedure, such as reliable cell counting, viable cell recovery, enzymatic digestion, and the requirement of an extracellular matrix as a scaffold. Furthermore, we showcase the potential of personalized drug testing as a promising application of GBM TOs. In conclusion, we successfully developed a robust workflow that effectively utilizes the limited material derived from stereotactic biopsies to reproducibly form standardized TOs. Moreover, we demonstrate that biopsy-derived TOs represent a valuable tool for testing drug vulnerabilities in a personalized setting, which might be especially useful in the case of non-resectable GBM.

## 1. Introduction

*IDH*-wildtype glioblastoma (GBM) represents the most common malignant and aggressive form of brain tumor in adults, with an extraordinary high mortality [1]. Its treatment options with clinical benefit are limited, and the standard of care is a maximal safe surgical resection followed by fractionated radiotherapy combined with adjuvant systemic admission of temozolomide [2]. However, due to the invasive nature of GBM, complete surgical resection is virtually impossible, and almost every GBM patient faces an inevitable risk of tumor progression and recurrence after the first line of therapy [3]. Thus, GBM remains incurable, resulting in a very poor prognosis, with a median overall survival of less than 15 months and a 5-year survival rate of only 6.8% [1,4,5].

Unfortunately, therapeutic interventions can also promote the expansion of treatment-resistant tumor cells to clonal evolution. This can result in altered genotypes and phenotypes of the recurrent tumor, which may significantly differ from the primary malignancy [6,7]. As a consequence, with disease progression, tumors can acquire an increased resistance against radiation and systemic treatment modalities, including temozolomide, which further limits the effective use for recurrent GBM [8,9]. Moreover, at this stage of the disease, surgical resection is often not feasible anymore due to the close proximity of the recurrent tumor to critical anatomical locations in the brain.

For such non-resectable tumors, frame-based stereotactic biopsies offer a minimally invasive, less risky technique for collecting tissue samples to enable accurate tumor diagnostics [10,11,12]. During this procedure, only very small amounts of tumor tissues are sampled from a predetermined localization within the tumor bulk. Apart from the diagnostic perspective, this kind of sample acquisition may offer the great opportunity to use this material to study responses to selected drugs based on therefrom-generated ex vivo models.

In addition to traditional pre-clinical models, such as two-dimensional (2D) monolayer and three-dimensional (3D) spheroid tumor cell cultures or mouse models [13,14,15,16,17,18], patient-derived tumor organoids (TOs) represent an exciting new technique in brain tumor research [19,20]. Importantly, in several previous studies, patient-derived GBM TOs, generated from minced tumor tissue or tumor-derived single cells, were shown to reflect the high complexity of the parental tumor by preserving the diverse tumor cellular states and tumor microenvironment (TME), and can be prepared within a few days [20,21,22,23]. Therefore, TOs not only harbor the potential to investigate patient-individual tumor characteristics, including present tumor cell clones, TME, and tumor–TME interactions in a 3D structure, but can also serve as a model system for studying drug-specific treatment responses ex vivo in a personalized and timely manner.

This study presents an optimized workflow for successfully generating patient-derived GBM TOs using the limited material derived from stereotactic biopsies. Furthermore, we highlight the critical steps during this procedure, such as cell counting, enzymatic digestion of the patient tumor, and the use of a basement membrane matrix to ensure an efficient and optimal TO formation even from this low amount of starting material. Notably, we further demonstrate the feasibility of using biopsy-derived TOs for personalized drug testing as a promising application in the field of precision oncology.

## 2. Materials and Methods

### 2.1. Patient Samples

Human tumor samples of *IDH*wt newly diagnosed and recurrent GBM were received from patients undergoing a frame-based stereotactic biopsy or open resection at the Department of Neurosurgery at the University Hospital Heidelberg, Germany. Simulated biopsies were prepared from open resections ex vivo by cutting the resected tumor from contrast-enhancing regions with a scalpel into 1 mm pieces. Informed consent was obtained from all patients. The Written Institutional Review Board of the Medical Faculty of Heidelberg approved the use of the patient material (ethic vote 005/2003 and S-672/2023).

### 2.2. Preparation of Single-Cell Suspensions from Biopsy Material

For the generation of tumor-derived single-cell suspensions, tumor biopsies were transferred to HBSS buffer (Life Technologies, Carlsbad, CA, USA) in a C-tube (Miltenyi Biotec, Bergisch Gladbach, Germany), containing Liberase (DH Research Grade, Roche, Darmstadt, Germany) and DNase I (Sigma-Aldrich, Taufkirchen, Germany). The enzymatic tumor dissociation into a single-cell suspension was performed using the gentleMACS Octo Dissociator with heaters (Miltenyi Biotec, Bergisch Gladbach, Germany). After digestion, the enzymatic reaction was stopped with 0.5% EDTA diluted in HBSS buffer, and the resulting cell suspension was filtered through cell strainers with a pore size of 100 µm and 40 µm to remove tissue debris. Finally, cells were resuspended in PBS to determine the cell number of the single-cell suspension via manual and automated cell counting.

### 2.3. Trypan-Blue Based Cell Couting

To determine cell numbers of the single-cell suspensions, we used a trypan blue-based cell counting method. The trypan blue exclusion dye penetrates the impaired cell membrane of dead cells, thus allowing viable cells to be distinguished from dead cells stained in blue within the single-cell suspension. For this purpose, the single-cell suspension was mixed 1:1 with the trypan blue dye and then applied either to a Neubauer counting chamber for manual cell number validation by eye or to a respective counting slide for automated cell counting with the TC20 Automated Cell Counter (Bio-Rad Laboratories, Berkley, CA, USA). The automated cell counter device offered the option to restrict the minimal cell size to 6 µm diameter and, therefore, to exclude smaller debris particles within the single-cell suspensions in a standardized way.

### 2.4. Cell Counting by Dual-Fluorescent Labeling

Another method of determining the cell number within the single-cell suspensions was based on dual-fluorescent cell labeling. The dual-fluorescent detection of nucleated cells was done via the addition of acridine orange (labeling viable cells) and propidium iodide stain (labeling dead cells) and was subsequently evaluated using the LUNA fl Dual Fluorescence Cell Counter (Logos Biosystems, Anyang-si, Republic of Korea) following the manufacturer’s instructions. In addition, cell size was restricted to a minimum of a 6 µm diameter to exclude autofluorescent particles and debris within the single-cell suspension.

### 2.5. Formation of Patient-Derived GBM TOs

To prepare patient-derived GBM TOs, freshly prepared single-cell suspensions were seeded at a defined cell number in a total volume of 50 µL (384-well format) or 100 µL (96-well format) culture media into low-attachment cell culture plates. Further, to increase the efficiency of the GBM TO formation, different concentrations of either Matrigel Matrix Basement Membrane (Matrigel, Corning, New York, NY, USA) or Cultrex Reduced Growth Factor Basement Membrane Extract Type 2 (BME-2, R&D Systems, Minneapolis, MN, USA) were added to the cells as a matrix scaffold to support cellular reaggregation. Cells were subsequently centrifuged for 5 min at 500× *g* and further cultivated at 37 °C and 5% CO_2_.

### 2.6. Bright-Field and Fluorescent Imaging of Patient-Derived GBM TOs

Bright-field images were taken to monitor GBM TO formation over time using the Olympus CKX53 microscope modified with the LC35 camera (Olympus, Tokyo, Japan). Image acquisition was performed with the Olympus cellSense Entry software (version 4.1). After TO formation, cell viability of GBM TOs was evaluated using the LIVE/DEAD Cell Imaging Kit (Invitrogen, Karlsruhe, Germany) following the manufacturer’s instructions. The fluorescent staining was imaged with the Olympus IX51 microscope equipped with an XM10 camera (Olympus), and fluorescent images were acquired using the Olympus cellSense Dimension software (version 1.17).

### 2.7. Assessment of TO Formation

To monitor successful TO formation, cell reaggregation and compactness were assessed by measuring the TO area over time in culture using ImageJ (version 1.54d, National Institute of Health, USA). To this end, the sizes of TOs on each day were normalized to the respective sizes of TOs on d0 for each condition and following over time.

### 2.8. Drug Testing on Patient-Derived GBM TOs

After successful TO formation, GBM TOs were used to study the sensitivity towards selected anti-cancer drugs. For this purpose, GBM TOs were treated for 72 h with selected drugs applied at different concentrations in technical triplicates to generate dose–response curves. After treatment, cell viability was determined by using the CellTiter-Glo 3D Cell Viability Assay (Promega, Walldorf, Germany) following the manufacturer’s instructions. The emerged luminescence was measured by detecting the relative light units (RLUs) using a microplate reader (Infinite F200 pro, Tecan, Männerdorf, Switzerland). Data were analyzed using the GraphPad Prism software (version 9.3.1, Diego, CA, USA). Raw data were normalized to the mean RLU values of 0.1% DMSO-treated TOs, which serve as negative control. For final analysis, determined cell viabilities were plotted against the log_10_ concentrations of the respective drug. Dose–response curves were generated using a normalized non-linear regression, and thereby, the half-maximal inhibitory concentrations (IC_50_) and the area under the curve (AUC) were assessed for each patient.

## 3. Results

**Figure 1:**
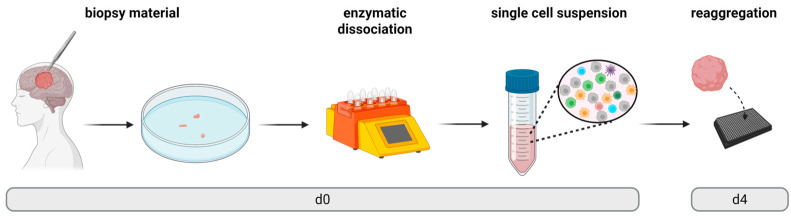
Workflow. Schematic overview of the experimental workflow for the generation of GBM TOs derived from stereotactic biopsies. On day 0, tissue samples were enzymatically dissociated into single-cell suspensions, which were subsequently seeded at a defined cell number into low-attachment microplates to allow reaggregation and TO formation until day 4. Abbreviations: TO = tumor organoid, d = day.

This study aimed to develop an optimized workflow for the generation of GBM TOs based on the limited material from frame-based stereotactic biopsies, enabling the assessment of patient-individual drug responses ex vivo (Figure 1). For this purpose, tissue biopsies were enzymatically dissociated into single-cell suspensions, which were then seeded at defined cell numbers into low-attachment microplates to promote the reaggregation of cells over time in vitro. Under optimal conditions, this procedure results in the successful formation of standardized TOs of equal size and cellular composition as the parental tumor [20].

However, given the limited amount of biopsy material, successful and standardized TO formation as a pre-requisite for drug response testing requires optimized experimental conditions, e.g., including the accurate determination of the number of viable cells and appropriate parameters for cell seeding and culturing. Therefore, in the following sections, we present the optimization of some of the most critical steps in the protocol in detail, which we suspected to impact successful formation and can help to improve the overall number of standardized TOs from limited biopsy material.

### 3.1. Fluorescent-Based Automated Cell Counting Ensured the Most Accurate Assessment of Viable Cell Numbers

The quality of the prepared single-cell suspension strongly depends on the original tissue architecture, and a high content of necroses is a hallmark of GBM. Therefore, the success of tumor dissociation in generating viable, pure single-cell suspensions can be specifically disturbed by cellular debris and a varying number of dead cells, as well as non-nucleated erythrocytes in GBM. Thus, assessing the correct number of viable cells in the single-cell suspension is a fundamental step to ensure an unbiased formation of standardized TOs of equal size.

**Figure 2:**
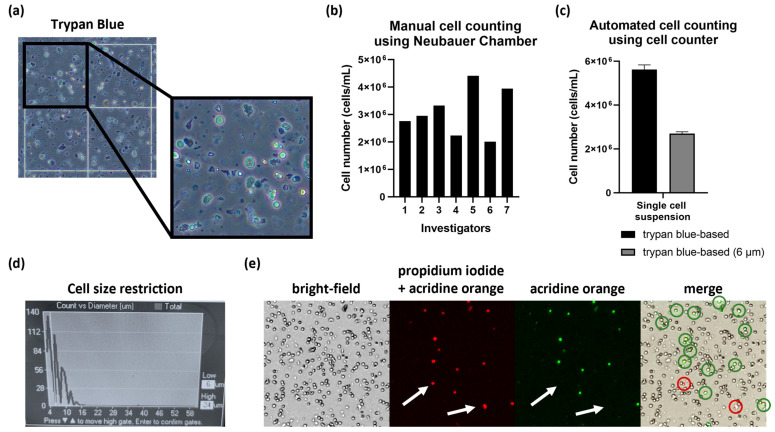
Cell counting optimization of nucleated cells. (**a**) Trypan blue staining visualized by a Neubauer counting chamber. (**b**) Manual cell counting by various individual investigators (1–7) using trypan blue staining and a Neubauer counting chamber showed high variations within the same single-cell suspension. (**c**) Cell counting based on trypan blue staining using an automated cell counter. (**d**) The minimum cell size can be limited to 6 µm to exclude smaller debris particles. (**e**) Cell counting using a dual-staining fluorescent-based automated cell counter uses propidium iodide labeling of the dead cells in red and acridine orange labeling of the cells in green (intercalation into double-stranded DNA) and red (single-stranded DNA and RNA). Only double-stained cells were detected as viable cells (green circles in merge), whereas the dead cells were solely labeled in red (marked by white arrows and red circles in merge).

Manual cell counting using the Neubauer counting chamber is still a frequently used counting procedure, which is based on staining with trypan blue, a dye that is incorporated exclusively into dead cells (Figure 2a). However, trypan blue does neither label living cells nor cellular debris, which impairs counting solely viable cells and leads to substantial inter-investigator variations, i.e., by identifying varying amounts of debris as viable cells, or viable cells as dead cells (Figure 2b). This is most likely due to extraordinarily high numbers of debris particles present after dissociation of tumor biopsies and unwanted staining of viable cells over time.

To improve reproducibility between various investigators and single-cell suspensions, it is also possible to validate the trypan blue staining by an automated device, resulting in more standardized cell counting (Figure 2c). Additionally, automated cell counters can be used to restrict the minimum cell size to 6 µm, thus excluding highly standardized smaller debris particles, which is less feasible with the naked eye (Figure 2c,d).

Nevertheless, the use of an automated cell counter with this mode of action may result in the inclusion of larger debris and erythrocytes present within the single-cell suspension, thereby hindering the precise assessment of the exact cell numbers within these suspensions. To account for this weakness, we tested an automated cell counter that is based on dual fluorescent staining of nucleated cells (Figure 2e). In this staining procedure, propidium iodide (PI) is applied as a non-permeable dye, which can solely penetrate non-viable cells with a leaky cell membrane, labeling nucleated dead cells in red. The second dye, acridine orange, penetrates into nucleated viable cells through their intact cell membrane and intercalates into double-stranded DNA, resulting in a green fluorescent signal, as well as into single-stranded DNA or RNA, resulting in a red fluorescent signal. As a consequence, nucleated viable cells emerge as double positive with both green and red fluorescence based on acridine orange stain, whereas dead cells are solely labeled by the PI-based red fluorescence, while non-nucleated erythrocytes and debris remain unstained. This technique ensures a more precise and reliable cell number assessment, which also reduces possible inter-user variations. Moreover, the device provides the cell counting results in images, thus enabling a visual inspection and validation of the counting results to ensure high accuracy.

### 3.2. Reduction of Enzymatic Digestion Time Did Not Impact Cell Number and Cell Viability During Tumor Dissociation

In addition to precise cell counting of viable cells within a single-cell suspension, optimizing the tumor dissociation is crucial to retrieve a maximum number of viable cells from the limited starting material without overdigestion, i.e., thereby potentially negatively impacting cell viability. Therefore, we investigated whether a reduction in incubation time of the enzymatic digestion during tumor dissociation of comparable small biopsies could positively affect the cell number and viability of cells.

**Figure 3:**
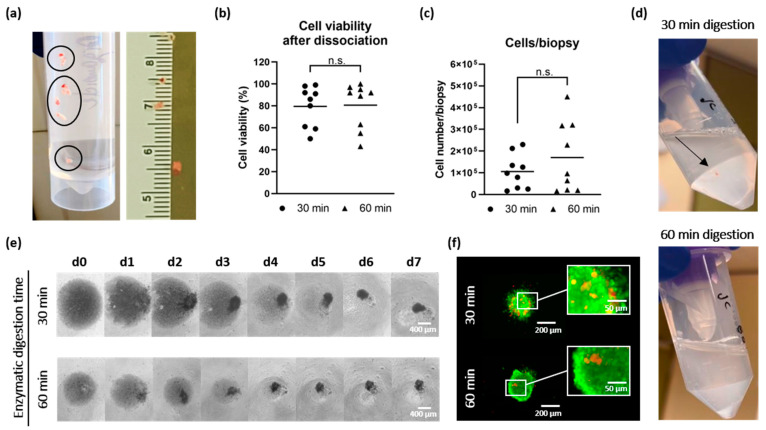
Effect on the enzymatic digestion time during tumor dissociation. (**a**) Two to eight biopsies were obtained per patient (encircled in black), measuring approximately 1 mm in diameter. (**b**) Mean cell viability showed no significant differences between 30 min and 60 min, while (**c**) mean cell number retrieved per biopsy tended to be increased in favor of 60 min (n = 9 cases) but was not significantly different. (**d**) Digestion of the biopsy material after 30 min (top) was incomplete, and the suspension still contained visible fragments (arrow) compared with digestion of 60 min (bottom). (**e**) TO formation of cells obtained from both digestion times was successful, which is also demonstrated by (**f**) live/dead staining on d7. For statistical analysis, paired *t*-tests were performed. Abbreviations: TO = tumor organoid, d = day, n.s. = not significant.

To this end, we used in total 2 to 8 stereotactic biopsies per patient with an approximate diameter of 1 mm (Figure 3a). The material was equally split into two samples to test different digestion times. The two samples were enzymatically digested in parallel using the gentleMACS dissociator for either 60 min, as recommended by the manufacturer’s instructions and as successfully used by us in the past for larger amounts of tumor material [20], or for only 30 min. We hypothesized that the reduction in the digestion time to 30 min may improve cell viability and decrease cell damage.

Interestingly, the digestion time did not significantly impact the results for both cell viability and cell number, based on biopsy samples of n = 9 GBM patients (Figure 3b,c). However, we found that the mean cell number per biopsy was even slightly increased following the manufacturer’s protocol of 60 min enzymatic digestion time, ranging from 16,000 to 450,000 isolated cells per biopsy compared to a maximum of 212,000 isolated cells per biopsy when the digestion process was stopped after 30 min, suggesting incomplete dissociation of cells after 30 min. This was also accompanied by the observation of a more homogenous suspension after 30 min of digestion (Figure 3d). The cell viability of the single-cell suspension ranged from 50% to 95% and appeared to be more dependent on the individual architecture of the input tumor material rather than on the enzymatic digestion time. Nevertheless, the success of TO formation was not negatively affected by the shorter digestion time, which was also demonstrated by live/dead staining of TOs after seven days (Figure 3e,f).

Taking these results together, even from small biopsies, the digestion time of 60 min did not have a negative impact on cell viability and TO formation, and was even required to obtain the maximum number of viable cells. Therefore, we decided to proceed with the enzymatic tumor digestion using the 60 min protocol to ensure a thorough tumor dissociation of the tumor biopsies and, consequently, to minimize the risk of any potential loss of viable cells.

### 3.3. Scaffold Concentration Is Important for GBM TO Formation

Despite optimized cell counting and digestion time, the resulting cell number still remained relatively low, ranging from 16,000 to 450,000 isolated cells per biopsy due to the low input material obtained from stereotactic biopsies (<0.1 g). Therefore, biopsy-derived TOs were prepared from a comparable low number of cells per well (10,000 cells) to increase the number of GBM TOs available for further drug testing. Since it cannot be excluded that a lower cell number impairs TO formation, we tested the use of basement membrane-like matrix scaffolds to support TO compaction, a characteristic we have previously found to indicate their successful formation. For this purpose, we added two different basement membrane-like matrix scaffolds at different concentrations to each well to assess which condition would optimally support TO formation.

**Figure 4:**
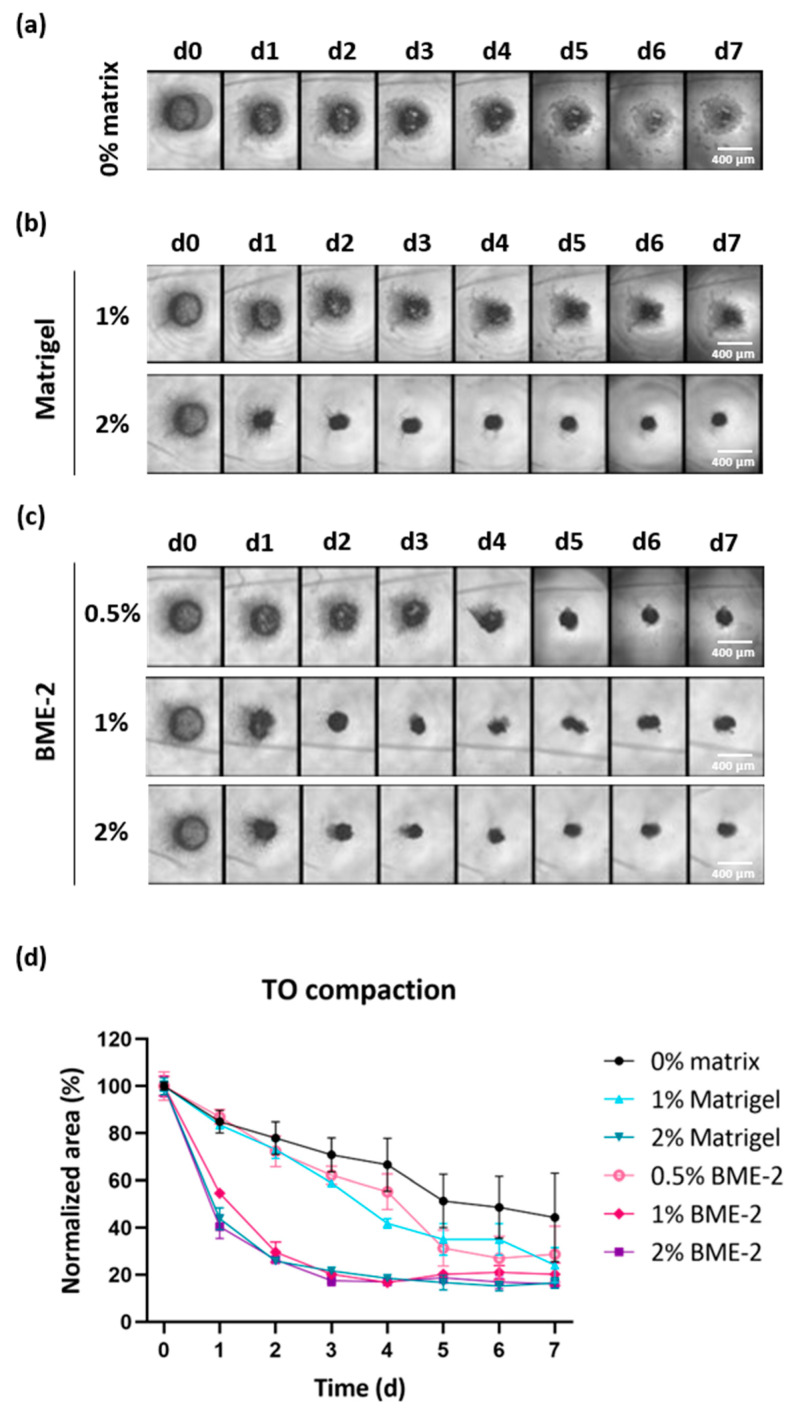
TO formation using different concentrations of scaffolds. TO formation was monitored by taking bright-field images in 4× magnification from d0 to d7 (**a**) in the absence of additional scaffolds or in the presence of (**b**) 1% and 2% Matrigel, or (**c**) 0.5%, 1%, and 2% BME-2. (**d**) TO compaction was determined by normalizing the measured TO area to the initial values on d0, including three TOs per condition (n = 1). Whiskers depict SD. Abbreviations: TO = tumor organoid, d = day, SD = standard deviation.

To this end, we seeded 10,000 cells per well (384-well) and added either no matrix or a defined concentration of Matrigel or BME-2 to GBM single cells. TO formation was monitored via bright-field images over time (Figure 4a–c and Figure S1a,c). Additionally, the degree of TO compaction was evaluated by quantifying the area of the developing TOs in bright-field images using ImageJ and the obtained values were normalized to the area observed at d0 for each experimental condition (Figure 4d and Figure S1b,d).

Regarding a representative case, in the absence of a matrix scaffold, a 55% reduction in the TO area was observed on d7, while the TO structure remained relatively loose and fragile (Figure 4a,d). The use of 1% Matrigel accomplished a 75% reduction on d7 in comparison to d0 (Figure 4b,d). However, the TO structure still seemed to be composed of more loosely connected cells, while using 2% Matrigel substantially improved this and led to a faster reduction in the initial TO area but resulted in a similar area on d7. When using BME-2 at a lower concentration of 0.5%, this resulted in a 71% reduction in the TO area within seven days (Figure 4c,d). However, by applying higher concentrations of 1% or 2% BME-2 per well, the reduction was increased to 79% and 84%, respectively. Regarding the formation speed, TOs started to form already after one day of incubation in the presence of 2% Matrigel, 1% BME-2, or 2% BME-2, leading already to 56%, 45%, and 60% reductions in TO area (Figure 4d). As for the latter conditions, TO formation already reached the ultimate compaction after four days for 2% Matrigel and even after three days for 1% and 2% BME-2. For the TO with no scaffold matrix and for 1% Matrigel and 0.5% BME-2, a slower compaction was observed. In general, the impact of the scaffold appeared to be case-dependent, whereby in all n = 3 cases, 2% BME-2 performed efficiently, as well as 1% BME-2 and 2% Matrigel (Figure S1).

In conclusion, these results demonstrate that 2% BME-2 supported the process of a quick and compact TO formation best, followed by 2% Matrigel and 1% BME-2.

### 3.4. Further Reduction in Cell Numbers per TO or Change in the Microplate Format Did Not Affect the Success of TO Formation

**Figure 5:**
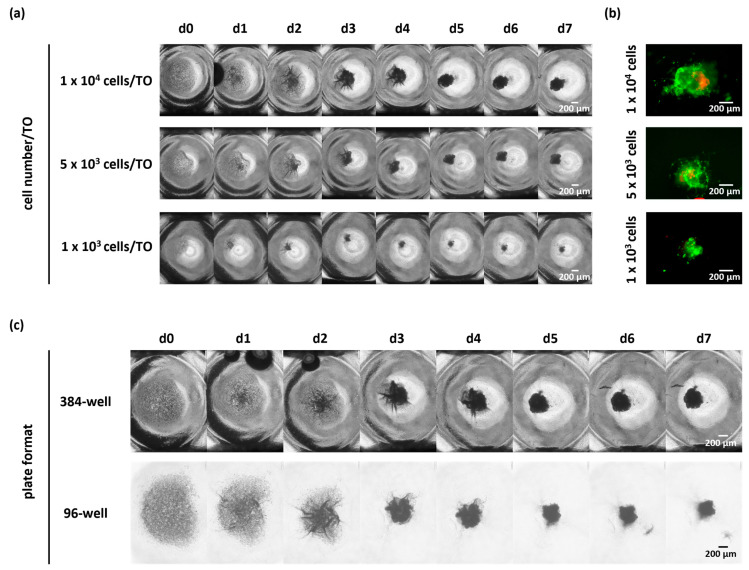
TO formation using different cell numbers and microplate format. TO formations (**a**) using different cell numbers (1 × 10^3^, 5 × 10^3^, 1 × 10^4^ cells per TO), (**b**) confirming viability by live/dead staining on d7 (green as viable cells, red as dead cells), and (**c**) comparing 384-well and 96-well low-attachment microplates, revealed successful TO formation for all conditions tested, as assessed by bright-field imaging over time. Abbreviations: TO = tumor organoid, d = day.

As shown before, the seeding of 10,000 cells per well resulted in a successful TO formation. Due to the low number of cells retrieved from biopsies, we next asked whether the cell number seeded per TO can be further reduced without impacting TO formation. Therefore, we seeded 5000 and 1000 viable cells in a low-attachment microplate as compared to 10,000 cells per well (Figure 5a,b and Figure S2). By monitoring the compaction of TOs via bright-field images, we observed successful cellular reaggregation over time regardless of the tested cell numbers in n = 3 cases (1000/5000/10,000 cells per TO).

In addition, we tested the impact of different plate formats, including 96-well and 384-well plates, on TO formation, since some applications may need different seeding volumes, and an adverse effect of different plate formats on TO formation could not be excluded due to an increased distance between cells in wells of larger sizes and the demand of higher medium volumes. To this end, we seeded 10,000 cells per well either in 384-well or 96-well microplates together with 2% BME-2 and cultivated the cells for up to seven days. TO formation was again monitored by bright-field images taken daily and demonstrated successful TO formation with 10,000 cells per TO in both microplate formats for n = 3 cases (Figure 5b and Figure S3).

Altogether, these results demonstrate that successful TO formation using our optimized protocol is even possible with lower cell numbers and independent of the plate format. This allows for a standardized GBM TO formation, even from the limited tumor material of biopsies.

### 3.5. Biopsy-Based Personalized Drug Testing on Patient-Derived TO

**Figure 6:**
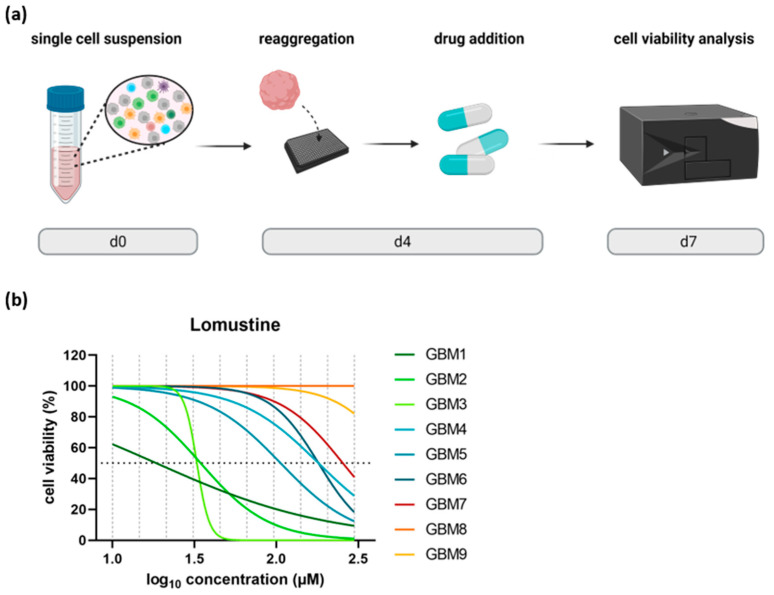
Personalized drug testing on patient-derived GBM TOs as a use case for stereotactic biopsies. (**a**) A total of 10,000 cells of freshly prepared single-cell suspensions were seeded into 384-well microplates together with 2% BME-2 to form TOs within four days. On d4, ten different concentrations were applied to TOs and cultivated for 72 h. On d7, the cell viability of treated TOs was determined by measuring luminescence. (**b**) Drug dose–response curves for lomustine were generated for n = 9 different GBM patients (GBM1–GBM9), testing ten different concentrations from 10 to 300 µM (marked with the vertical grey dashed lines). The various curve profiles represent the heterogeneity of the response of individual patients to the respective drug. Abbreviations: TO = tumor organoid, d = day, GBM = glioblastoma.

Finally, we tested if our optimized workflow to generate standardized GBM TOs starting from the limited tumor material obtained from stereotactic biopsies can be used as an ex vivo model system to reliably study individual drug responses. To this end, TO-based drug response was assessed for n = 9 GBM patients (Figure 6b). A total of 10,000 cells of freshly prepared single-cell suspensions were seeded per well with 2% BME-2 in a 384-well plate to allow for TO formation. A compact TO formation was achieved within four days (Figure 6a). On d4, lomustine, a DNA alkylating agent commonly used for recurrent GBM [24], was applied in technical triplicates to the TOs in ten concentration steps, ranging from 10 to 300 µM, and incubated for 72 h. Subsequently, cell viability was measured via cell viability assay that determines the ATP production of cells.

Obtained drug response curves showed a broad sensitivity range demonstrated by the calculated IC_50_ and AUC values. The calculated IC_50_ values varied from 18 µM to more than 250 µM (Table 1). For two GBM patients, IC_50_ values could not be reached within the concentration range tested. Moreover, AUC values showed also a wide distribution reaching from 46.6 ± 2.7 to 195.6 ± 4.0. The high variability observed between the individual cases reflects the known intertumoral heterogeneity of GBM patients and indicates that TOs generated from limited biopsy material represent a valuable tool to mirror patient-individual drug responses. Therefore, this technique could be an important pillar towards precision medicine, especially in dealing with limited biopsy material.

## 4. Discussion

Precision medicine, with the aim of identifying the most effective treatment for each cancer patient, has the potential to improve their outcome significantly. One of the major challenges is the accurate prediction of individual drug sensitivities, for instance, by using patient-derived TOs. In particular, the therapeutic options for recurrent GBM patients are still limited and largely inefficient. In addition, at this stage of the disease, maximal surgical resection is frequently no longer possible due to the patient’s decline or the location of the tumor. For this advanced disease stage, frame-based stereotactic biopsies offer a less invasive and safer method of obtaining at least tumor tissue for diagnostic purposes and, additionally, may provide the possibility of testing responses to selected drugs in vitro [10,11,12]. However, to the best of our knowledge, the feasibility of using biopsy material for drug screening has not been explored, while its successful use for other applications, such as integrated multi-modal molecular analyses, has been reported previously [25]. Unfortunately, TO-based drug testing is even more challenging due to the limited tissue amounts obtained by frame-based stereotactic biopsies. The major drawbacks are the number of viable cells as input material for the preparation of TOs, as well as reliable counting and formation of TOs. This finally determines the number of drugs that can be tested.

In the present study, we optimized some of these critical steps by providing a method for accurate cell counting of viable cells, besides a highly standardized exclusion of debris and dead cells within a single-cell suspension. We further tested an efficient recovery of viable cells during tumor digestion and different types of cell seeding modalities. The resulting protocol led to a standardized and robust workflow, allowing for the successful preparation of TOs of a similar size within a few days. Within this short time window, TO formation and subsequent ex vivo personalized drug testing of selected drugs is possible in a clinically relevant timeframe. Furthermore, the risk of acquiring additional molecular genetic alterations in tumor cells or the loss of stromal cell populations because of prolonged cultivation is minimal within the chosen time window of one to two weeks [22]. Moreover, this fast 3D standardized mini-tumor model ensures the representation of intratumoral cellular heterogeneity, with the TME being preserved within the TO, as demonstrated in our recent work [20]. Moreover, we have shown that TO formation does not only result in a tumor tissue-like architecture, but also maintains the mutational and transcriptional profile of the parental tumor [21]. Although some publications already have shown the successful formation of patient-derived GBM TOs starting from material obtained from open resections [19,22,23,26], a protocol for the challenging use of GBM-derived biopsy material to successfully prepare patient-derived TOs provides an important improvement in the application range.

We have shown that the cell counting of single-cell suspensions is one of the crucial steps for standardized TO formation since it can be error-prone. As demonstrated, manual counting would have introduced a significant bias into the enumeration of viable cells due to the substantial inter-investigator variations in cell counts, with some investigators reporting numbers that were 40% lower and others reporting numbers that were 50% higher than the median number of cells detected by all investigators (n = 7). Consequently, this would create a vast bias in TO size, especially for subsequent experiments, making reliable and reproducible cell counting indispensable to standardize TO formation. We have solved this issue using a fluorescent-based cell counting device using acridine orange and propidium iodide staining, allowing the positive selection of viable nucleated cells and providing results in a comprehensive and traceable way. Several other studies also underline the importance of correct and reliable cell counting [27,28,29,30]. For instance, Chan et al. used freshly isolated PBMCs to compare the different cell counting methods, including trypan blue applied combined with manual cell counting, and acridine orange and propidium iodide dual staining with an automated cell counting system [30]. Notably, the authors demonstrated that the use of the fluorescent-based staining with acridine orange and propidium iodide reduced the bias caused by erythrocytes included in the cell suspensions since the method targets solely nucleated cells. As our biopsy-derived cell suspensions contain, in addition to erythrocytes, a large quantity of cellular debris resulting from the tumor digestion, automated counting of viable cells with the help of two dyes constitutes a further important advantage.

Furthermore, we have demonstrated that an extracellular matrix as a scaffold is of great help to obtain a rapid and reliable TO formation. Interestingly, both tested matrices, Matrigel and BME-2, were found to be applicable in this protocol. However, the concentration had to be optimized and a 2% solution of either BME-2 or Matrigel seemed to perform best.

Another critical step has been to test how much the number of viable cells used for TO formation can be reduced without disturbing the compaction of the TO-like structure. This allows the preparation of more TOs for personalized drug testing out of the low input material of small biopsies. In addition, the possibility of using 96-well plates and 384-well plates for TO formation without any obvious disadvantages facilitates further adaptations of the application format, which might be helpful for distinct drug formulations and experimental setups.

In conclusion, we have successfully established a workflow for effectively utilizing the low starting material from frame-based stereotactic tumor biopsies to generate patient-derived GBM TOs. Importantly, we have demonstrated that the use of accurate cell counting and the optimized use of scaffolds enable the reliable formation of standardized TOs, and TOs of similar size, which further represents an important ex vivo tool for testing patient-specific treatment responses within a short time window, and thus providing a unique and promising experimental therapeutic approach for GBM patients with limited material availability.

## Figures and Tables

**Table 1:** Anti-proliferative effects of lomustine upon treatment of TOs derived from n = 9 GBM patients, expressed as IC_50_ (µM) and AUC values with SD.

GBM Patient	IC_50_ (µM)	AUC
GBM1	18.5	46.6 ± 2.7
GBM2	34.5	50.3 ± 7.4
GBM3	32.7	52.3 ± 4.6
GBM4	180.9	120.1 ± 3.9
GBM5	106.2	93.2 ± 5.6
GBM6	180.9	116.8 ± 6.3
GBM7	254.8	137.7 ± 2.8
GBM8	n.d.	195.6 ± 4.0
GBM9	n.d.	164.1 ± 11.3

n.d. = not detectable.

## Data Availability

The original contributions presented in this study are included in the article/Supplementary Material. Further inquiries can be directed to the corresponding author(s).

## Supplementary Materials

The following supporting information can be downloaded at: https://www.mdpi.com/article/10.3390/cells14100701/s1, Figure S1: Comparison of patient-derived TO formation using various concentrations of Matrigel and BME-2. Figure S2: Impact of cell number on TO formation. Figure S3: Successful TO formation in both microplate formats.

## References

[1] Louis D.N., Ohgaki H., Wiestler O.D., Cavenee W.K., Burger P.C., Jouvet A., Scheithauer B.W., Kleihues P. (2007). The 2007 WHO classification of tumours of the central nervous system. Acta Neuropathol..

[2] Stupp R., Mason W.P., van den Bent M.J., Weller M., Fisher B., Taphoorn M.J.B., Belanger K., Brandes A.A., Marosi C., Bogdahn U. (2005). Radiotherapy plus concomitant and adjuvant temozolomide for glioblastoma. N. Engl. J. Med..

[3] Hatoum A., Mohammed R., Zakieh O. (2019). The unique invasiveness of glioblastoma and possible drug targets on extracellular matrix. Cancer Manag. Res..

[4] Marenco-Hillembrand L., Wijesekera O., Suarez-Meade P., Mampre D., Jackson C., Peterson J., Trifiletti D., Hammack J., Ortiz K., Lesser E. (2020). Trends in glioblastoma: Outcomes over time and type of intervention: A systematic evidence based analysis. J. Neurooncol..

[5] Ostrom Q.T., Price M., Neff C., Cioffi G., Waite K.A., Kruchko C., Barnholtz-Sloan J.S. (2022). CBTRUS Statistical Report: Primary Brain and Other Central Nervous System Tumors Diagnosed in the United States in 2015–2019. Neuro Oncol..

[6] Yalamarty S.S.K., Filipczak N., Li X., Subhan M.A., Parveen F., Ataide J.A., Rajmalani B.A., Torchilin V.P. (2023). Mechanisms of Resistance and Current Treatment Options for Glioblastoma Multiforme (GBM). Cancers.

[7] Gupta K., Burns T.C. (2018). Radiation-Induced Alterations in the Recurrent Glioblastoma Microenvironment: Therapeutic Implications. Front. Oncol..

[8] Nandeesh B.N., Naskar S., Shashtri A.H., Arivazhagan A., Santosh V. (2018). Recurrent Glioblastomas Exhibit Higher Expression of Biomarkers with Stem-like Properties. J. Neurosci. Rural Pract..

[9] Goenka A., Tiek D., Song X., Huang T., Hu B., Cheng S.-Y. (2021). The Many Facets of Therapy Resistance and Tumor Recurrence in Glioblastoma. Cells.

[10] Apuzzo M.L., Chandrasoma P.T., Zelman V., Giannotta S.L., Weiss M.H. (1984). Computed tomographic guidance stereotaxis in the management of lesions of the third ventricular region. Neurosurgery.

[11] Kondziolka D., Lunsford L.D. (1999). The role of stereotactic biopsy in the management of gliomas. J. Neurooncol..

[12] Ostertag C.B., Mennel H.D., Kiessling M. (1980). Stereotactic biopsy of brain tumors. Surg. Neurol..

[13] Gómez-Oliva R., Domínguez-García S., Carrascal L., Abalos-Martínez J., Pardillo-Díaz R., Verástegui C., Castro C., Nunez-Abades P., Geribaldi-Doldán N. (2020). Evolution of Experimental Models in the Study of Glioblastoma: Toward Finding Efficient Treatments. Front. Oncol..

[14] Haddad A.F., Young J.S., Amara D., Berger M.S., Raleigh D.R., Aghi M.K., Butowski N.A. (2021). Mouse models of glioblastoma for the evaluation of novel therapeutic strategies. Neurooncol. Adv..

[15] Fermi V., Regulska E., Wolfram A., Wessling P., Rominger F., Herold-Mende C., Romero-Nieto C. (2022). Luminescent Pyrrole-Based Phosphaphenalene Gold Complexes: Versatile Anticancer Tools with Wide Applicability. Chemistry.

[16] Dao Trong P., Jungwirth G., Unterberg A., Herold-Mende C., Warta R. (2023). The Antiepileptic Drug Oxcarbazepine Inhibits the Growth of Patient-Derived Isocitrate Dehydrogenase Mutant Glioma Stem-like Cells. Cells.

[17] Haydo A., Schmidt J., Crider A., Kögler T., Ertl J., Hehlgans S., Hoffmann M.E., Rathore R., Güllülü Ö., Wang Y. (2025). BRAT1—A new therapeutic target for glioblastoma. Cell. Mol. Life Sci..

[18] Reisbeck L., Linder B., Tascher G., Bozkurt S., Weber K.J., Herold-Mende C., van Wijk S.J.L., Marschalek R., Schaefer L., Münch C. (2023). The iron chelator and OXPHOS inhibitor VLX600 induces mitophagy and an autophagy-dependent type of cell death in glioblastoma cells. Am. J. Physiol. Cell Physiol..

[19] Hubert C.G., Rivera M., Spangler L.C., Wu Q., Mack S.C., Prager B.C., Couce M., McLendon R.E., Sloan A.E., Rich J.N. (2016). A Three-Dimensional Organoid Culture System Derived from Human Glioblastomas Recapitulates the Hypoxic Gradients and Cancer Stem Cell Heterogeneity of Tumors Found In Vivo. Cancer Res..

[20] Fermi V., Warta R., Wöllner A., Lotsch C., Jassowicz L., Rapp C., Knoll M., Jungwirth G., Jungk C., Dao Trong P. (2023). Effective Reprogramming of Patient-Derived M2-Polarized Glioblastoma-Associated Microglia/Macrophages by Treatment with GW2580. Clin. Cancer Res..

[21] Jungwirth G., Cao J., Lotsch C., Warta R., Moustafa M., Knoll M., Yu T., Braun V., Jassowicz L., Dao Trong P. (2024). Personalized Medicine for Meningiomas: Drug Screening on Tumor Organoids Exposes Therapeutic Vulnerabilities to HDAC1/2i Panobinostat. bioRxiv.

[22] Jacob F., Salinas R.D., Zhang D.Y., Nguyen P.T.T., Schnoll J.G., Wong S.Z.H., Thokala R., Sheikh S., Saxena D., Prokop S. (2020). A Patient-Derived Glioblastoma Organoid Model and Biobank Recapitulates Inter- and Intra-tumoral Heterogeneity. Cell.

[23] Golebiewska A., Hau A.-C., Oudin A., Stieber D., Yabo Y.A., Baus V., Barthelemy V., Klein E., Bougnaud S., Keunen O. (2020). Patient-derived organoids and orthotopic xenografts of primary and recurrent gliomas represent relevant patient avatars for precision oncology. Acta Neuropathol..

[24] Weller M., Le Rhun E. (2020). How did lomustine become standard of care in recurrent glioblastoma?. Cancer Treat. Rev..

[25] Yu K.K.H., Basu S., Baquer G., Ahn R., Gantchev J., Jindal S., Regan M.S., Abou-Mrad Z., Prabhu M.C., Williams M.J. (2023). Investigative needle core biopsies for multi-omics in Glioblastoma. medRxiv.

[26] Jacob F., Ming G.-L., Song H. (2020). Generation and biobanking of patient-derived glioblastoma organoids and their application in CAR T cell testing. Nat. Protoc..

[27] Cadena-Herrera D., Esparza-De Lara J.E., Ramírez-Ibañez N.D., López-Morales C.A., Pérez N.O., Flores-Ortiz L.F., Medina-Rivero E. (2015). Validation of three viable-cell counting methods: Manual, semi-automated, and automated. Biotechnol. Rep..

[28] Fagète S., Steimer C., Girod P.-A. (2019). Comparing two automated high throughput viable-cell counting systems for cell culture applications. J. Biotechnol..

[29] Johnston G. (2010). Automated Handheld Instrument Improves Counting Precision Across Multiple Cell Lines. BioTechniques.

[30] Chan L.L.-Y., Laverty D.J., Smith T., Nejad P., Hei H., Gandhi R., Kuksin D., Qiu J. (2013). Accurate measurement of peripheral blood mononuclear cell concentration using image cytometry to eliminate RBC-induced counting error. J. Immunol. Methods.

