# Supplementary material for: Use of Tissue Specimens from Stereotactic Biopsies for Patient-Derived GBM Organoid-Based Drug Testing
Source: Cells. 2025 May 12;14(10):701. doi: 10.3390/cells14100701 (PMC12109714; doi:10.3390/cells14100701)
Supplement: Supplementary file 1 [file cells-14-00701-s001.zip › cells-3597998-supplementary.pdf]

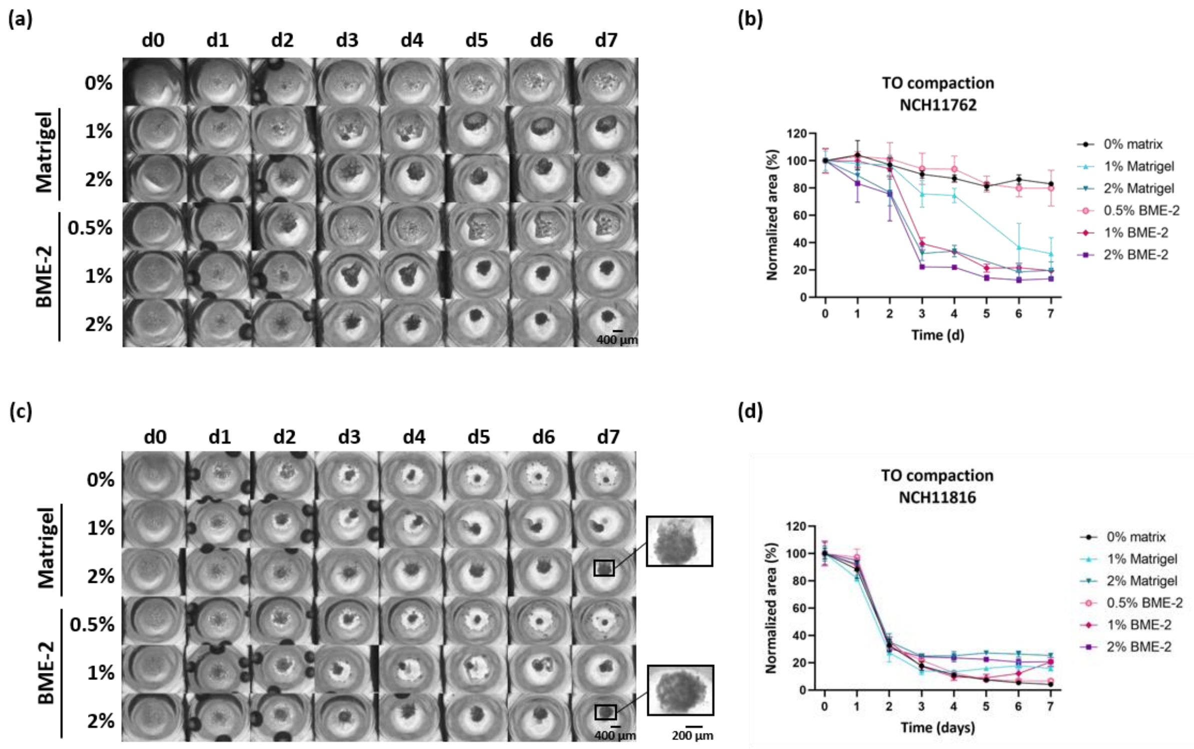

Figure S1. Comparison of patient-derived TO formation using various concentrations of Matrigel and BME-2. **(a**, **c)** shows TO formation of two distinct GBM cases, monitored by taking bright-field images from d0 to d7 following the protocol using either no scaffold matrix, 1% or 2% Matrigel, or 0.5%, 1%, or 2% BME-2. **(b**, **d)** The developing TO compaction was determined by normalizing the TO area compared to the initial values on d0, including three TOs per condition for the two respective GBM cases. Whiskers depict SD. Abbreviations: TO = tumor organoid, d = day, SD = standard deviation.

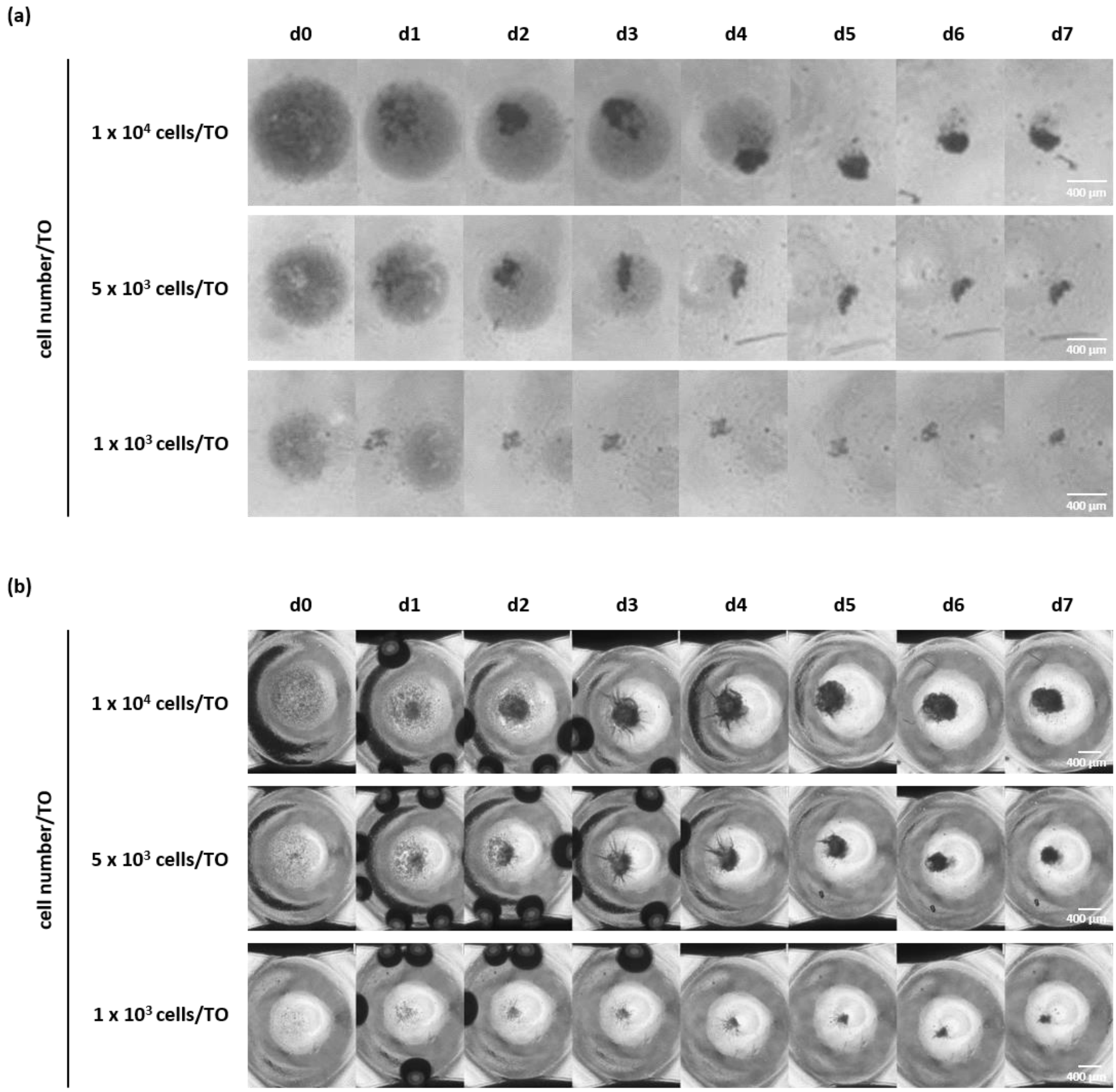

Figure S2. Impact of cell number on TO formation. TO formation using either 5 x 10^3 or 1 x 10^3 cells per well compared to the initial amount of 1 x 10^4 cells per well, was successful for all conditions tested, as assessed by bright-field imaging over time. Abbreviations: TO = tumor organoid, d = day.

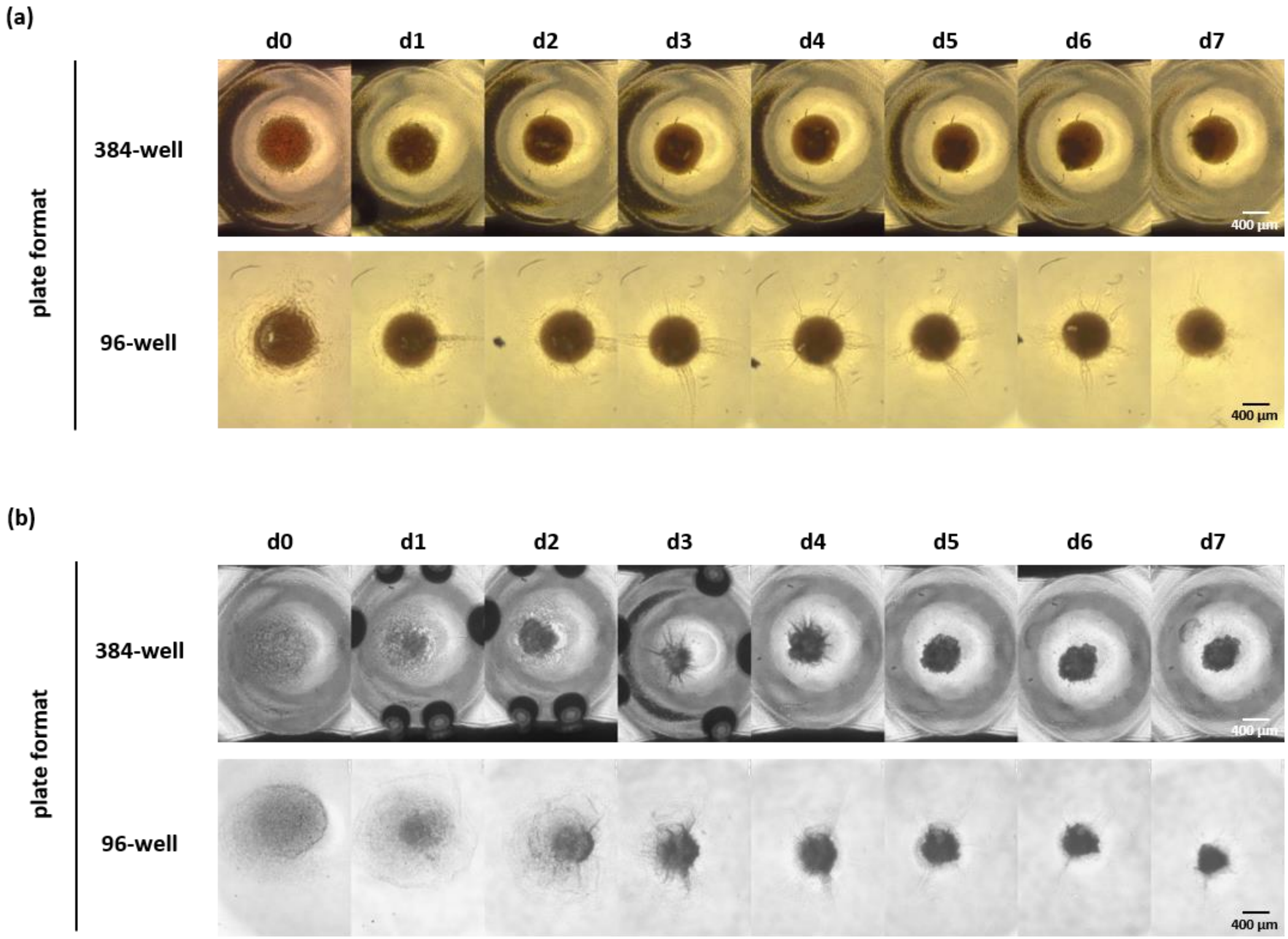

Figure S3. Successful TO formation in both microplate formats. TO formation, comparing the plate format using 384-well and 96-well low attachment microplates, was successful in both microplate formats, as assessed by bright-field imaging over time. Abbreviations: TO = tumor organoid, d = day.
